# T Cell Response Toward Tissue-and Epidermal-Transglutaminases in Coeliac Disease Patients Developing Dermatitis Herpetiformis

**DOI:** 10.3389/fimmu.2021.645143

**Published:** 2021-04-20

**Authors:** Marzia Caproni, Manuela Capone, Maria Caterina Rossi, Veronica Santarlasci, Laura Maggi, Alessio Mazzoni, Beatrice Rossettini, Daniela Renzi, Lavinia Quintarelli, Beatrice Bianchi, Alessandra Ninci, Gabriele Lami, Antonio Calabrò, Lorenzo Cosmi, Francesco Annunziato, Francesco Liotta

**Affiliations:** ^1^ Rare Diseases Unit, Section of Dermatology, Department of Health Sciences, USL Toscana Centro, University of Florence, European Reference Network-Skin Member, Florence, Italy; ^2^ Department of Experimental and Clinical Medicine, School of Human Health Sciences, University of Florence, Florence, Italy; ^3^ Department of Biomedical Experimental and Clinical Sciences “Mario Serio”, University of Florence, Florence, Italy; ^4^ Department of Health Sciences, University of Florence, Florence, Italy; ^5^ Medical Specialization School of Hygiene and Preventive Medicine, University of Florence, Florence, Italy

**Keywords:** T lymphocytes (CD4+), tissue transglutaminase (TG2), epidermal transglutaminase, celiac disease, cross reactivity

## Abstract

The reason why only few coeliac patients develop the cutaneous manifestation of the disease, named dermatitis herpetiformis (DH), is still unknown. Epidermal transglutaminase (TG3) has been described as the main autoantigen of humoral immunity in DH but the mechanisms leading to this autoimmune response remain obscure. Here we characterized T cells from skin, gut and peripheral blood of DH and coeliac disease (CD) patients, evaluated the impact of the gluten-free diet on circulating T lymphocytes’ phenotype and investigated antigen specific T cell response toward epidermal and tissue transglutaminase (TG2). DH patients showed an increased frequency of skin-derived T cells producing TNFα when compared to CD patients. Moreover, circulating T cells producing TNFα and IL-17A positively correlated with clinical score of skin disease activity and decreased after gluten-free diet. Finally, TG2 and TG3-specific T cells resulted more reactive to antigens stimulation in DH patients and showed cross reactivity toward the two autoantigens in both the group of patients. Our data suggest a role of TNFα and IL-17A producing cells in the development of DH and, for the first time, show the existence of a crossed T cell response toward the two transglutaminases isoforms, thus suggesting new insights on T cells role in skin damage.

## Introduction

Dermatitis herpetiformis (DH) is considered the specific cutaneous manifestation of celiac disease (CD). It consists of a chronic, polymorphic cutaneous eruption characterised by the granular deposition of IgA at the dermal papillae. Both DH and CD occur in gluten-sensitive subjects, showing a resolution of the clinical manifestations upon gluten withdrawing from the diet and a relapse when gluten is reintroduced (1). The pathogenesis of both the disorders is based on a complex interaction between environmental and genetic factors and on a cooperation of the innate and adaptive compartments of the immune system (2). DH and CD share the same predisposing Human Leukocyte Antigen (HLA) haplotype DQ2/DQ8 and the inflammatory T helper 1 (Th1)-type immune response against gluten that leads to the production of circulating IgA and IgG autoantibodies. Tissue transglutaminase (TG2) is a multifunctional tissue protein highly expressed in the small intestine and has been recognized as a major autoantigen of CD (3). The presence in CD patients’ sera of IgA autoantibodies directed to TG2 is crucial in the diagnosis of CD (4). This enzyme is strictly involved in CD pathogenesis because of its ability to modify gluten peptides by deamidation, which facilitates their presentation to the immune system *via* HLA-DQ2 or DQ8 molecules (5). Due to the lymphocytic infiltrate observed in the active CD intestinal lesions (6), and to the strong association of the CD with HLA class II molecules (7), the role of T-cells in the pathogenesis of CD has been long-established, also before the isolation of gliadin-specific T cells from the CD intestinal mucosa (8, 9).

The existence of TG2-specific T cells was confirmed in a study by Ciccocioppo et al., describing the presence and proliferation of CD4+ T cells, after stimulation with TG2, in an HLA-DQ2-restricted manner in the peripheral blood of untreated CD patients (10). While intestinal immune response to gluten has been widely studied either in term of the involvement of different T helper cell subsets and antigen specificity (11–16), the same did not occur for DH. In particular, the reason why only a subgroup of patients with CD develop DH is still unclear. Not all DH patients show the typical features of CD, such as a partial or total villous atrophy, but rather a modest lymphocytic infiltrate at the intestinal mucosa, with constant alteration of intestinal permeability (17). In 2002, Sardy et al. showed that TG3, an enzyme belonging to the same family of TG2 but expressed above all in the epidermis, was the main autoantigen of DH. However, the mechanism by which DH patients develop an autoimmune response against TG3 remains still obscure (18).One hypothesis concerns an epitope spreading phenomenon between TG2 and TG3; that share a high sequence homology (19). Zone et al. confirmed the role of anti-TG3 antibodies in the pathogenesis of DH: they transferred goat and human anti-TG3 IgG and IgA, respectively, into mice, demonstrating the presence of the same granular deposits at the dermal papillae found in patients with DH. However, mice did not show signs and symptoms of DH, suggesting that other mechanisms are necessary for the occurrence of the typical skin lesions (20). Moreover, in a mouse model of DH, that utilizes the NOD background and the HLA-DQ8 transgene, mice developed blistering pathology similar to that seen in DH but not the small-bowel manifestation (21). Another recent study demonstrated that DH patients produce anti-TG3 IgA in the small bowel suggesting that autoimmunity against TG3 is likely to occur in the gut (22). In parallel, the involvement of T cells in DH skin lesions has been hypothesized and investigated in few preliminary works. Garioch et al. found the presence of T cells, mainly CD4+, in DH lesions, suggesting the importance of a T cells mediated reaction in the generation of the skin damage (23). Our group described, by immunohistochemistry analysis, the hyper-activation of Th2-cells at skin level: Th2-specific cytokines along with those produced by granulocytes and macrophages, are able to recruit eosinophils, which co-operate with neutrophils to the cleavage of the dermo-epidermal junction. Moreover, we demonstrated a down-regulation of T-reg cells in the skin of DH patients suggesting a possible mechanism contributing to the maintenance of a pro-inflammatory tissue microenvironment (24, 25). In a study of Savilahti et al. the role of α/β and γ/σ T cells in DH has been studied: they demonstrated the presence and the high density of γ/σ T cells in the lamina propria and in the epithelium of jejunum in DH patients similar to those with CD. In addition, they showed that the levels of γ/σ T cells did not change after gluten free diet (GFD) (26–30). Finally, Baker et al. tried to demonstrate the presence of gluten-specific T cells lines in the skin of DH patients without any result (31).

The present study is aimed to characterize T cells in the skin, intestine and peripheral blood of both patients with active DH and CD patients without DH and monitor the T cell response in the blood during GFD. Finally, we will evaluate the antigen specific T cell response toward the two isoforms of tranglutaminase in DH patients compared to the CD patients’ one.

## Materials and Methods

### Dermatitis Herpetiformis (DH) and Celiac Disease (CD) Patients

The study was approved by the medical ethical committee of our Hospital (Azienda USL Toscana Centro, P.O. Piero Palagi, 663/2013/OSSCESM) no profit, and was conducted according to the Declaration of Helsinki.

Patients with DH were enrolled consecutively in Dermatology Department, Rare Diseases Unit, of the University of Florence from December 2014 to July 2018. Patients with CD were enrolled in the gastroenterology department of the University of Florence during the same period.

Diagnosis of DH was made on currently available diagnostic criteria which include detection with the direct immune-fluorescence (DIF) of specific granular IgA deposits at the dermal papillae associated with the presence of clinical and histopathological features suggestive for DH. None of the enrolled patients was on therapy and/or gluten-free diet (32).

At the same time, CD was diagnosed based on specific diagnostic criteria, such as a combination of clinical, serological and histopathological findings. None of the enrolled patients was on therapy and/or gluten-free diet.

Nineteen DH patients (7 males and 12 females; average age 45, 84 years; range 9-77), and 13 CD patients (7 males and 6 females; average age 36, 85; range 17–70 years) were enrolled in the study.

For each DH patient, personal data, clinical findings, HLA, duodenal and skin biopsies were collected (**Table 1**). The same data were collected for CD patients (**Table 2**).

**Table 1:** Clinical features of CD patients with DH.

				T0						
PT#	AGE	SEX	CLINICAL FINDINGS		SERUM BIOMARKERS		HLA	MARSH	HISTOPATHOLOGICAL FINDINGS	DIF
			Lesions	Sites	anti-TG2 (<9 neg 9-16 BL >16 pos)	anti-TG3 (<16 neg 16-22 BL >22 pos)				
**1**	10	F	erythemavesicles	scalp	49	21	ND	MARSH 3B	Focal epidermal exocytosis; perivascular inflammatory cell infiltrate in the superficial dermis	IgA ++ C3 ++
**2**	50	F	erythemaerosionpapules	elbowshands	> 100	343	ND	MARSH 3B	neutrophilicpapilitis	IgA ++ IgM + C3 +(+)
**3**	60	F	erythemapapules	elbowskneesbuttockpubis	9,4	73,1	DQ2 -DR3 *	MARSH 2	Mild perivascular inflammatory cell infiltrate dermal-epidermal detachment	IgA ++ C3 +(+)
**4**	67	M	erythemapapules	elbowsbackbuttockknees	> 100	125	DQ2 - DR3/DQ2 - DR7	MARSH 3B	epidermal acanthosis mild perivascular inflammatory cell infiltrate in the superficial dermis dermal-epidermal detachment	IgA ++ C3 +
**5**	27	M	erythemaerosionpapulesvesicles	elbowskneesbuttockbackpubisface	> 100	24,04	ND	MARSH 3C	hyperorthokeratosis, acanthosis mixed inflammatory infiltrate in the superficial dermis	IgA +(+) C3 +
**6**	54	M	erythemavesicles	elbowshandsankles	2,3	1,97	DQ2 -DR3 *	MARSH 0	Mild perivascular inflammatory cell infiltrate dermal-epidermal detachment	IgA + IgM + C1q +
**7**	60	F	erythemapapulescrust	elbowstrunkbuttocks	98	>150	DQ8 - DR4 *	MARSH 1	Mixed inflammatory infiltrate in the superficialdermis	IgA ++ C3 + C1q +
**8**	77	F	erythemapapules	elbowskneesbuttocks	15	97,3	ND	ND	neutrophilicpapilitis	IgA +(+) IgM + C3 +
**9**	43	F	erythemaerosionpapulesvesicles	elbowsknees	> 100	281	DQ2 - DR3/DQ8 - DR4	MARSH 3C	hyperorthokeratosis dermal-epidermal detachment neutrophilic papilitis mixed inflammatory cell infiltrate in the superficial dermis	IgA ++ C3 ++
**10**	37	F	erythemaerosionvesicles	elbowshandsankles	>100	158	DQ2 -DR3 *	ND	subepidermal blister with neutrophils located at the tips of the dermal papillae Perivascular inflammatory cell infiltrate.	IgA ++ IgG + C3 +
**11**	28	M	erythevesicles	elbowsknees	29,9	13,3	ND	ND	Subepidermal blister Mixed perivascular inflammatory infiltrate neutrophilic papilitis	IgA ++ IgG + C3 +
**12**	54	F	erythemapapulesvesicles	elbowsbuttocksabdomen	6,4	17,2	DQ2 - DR3/DQ2 - DR3	MARSH 0	dermal-epidermal detachment neutrophilic papilitis	IgA + IgM + C3 +
**13**	52	F	erythemapapulescrust	elbowskneesbuttocks	> 100	26,9	DQ2 -DR3 *	MARSH 3A	dermal-epidermal detachment with neutrophils accumulation in the superficial dermis	IgA ++ IgM + C3 +
**14**	39	F	erythemapapules	elbowskneesscalp	20	19,2	ND	ND	epidermal acanthosis mild perivascular inflammatory infiltrate in the superficial dermis	IgA ++ IgM + C3 +
**15**	42	F	erythemapapulesvesicles	elbowskneesbuttocksfeet	> 100	149,7	DQ2 - DR3/DQ2 - DR7	MARSH 3A	hyperorthokeratosis mixed inflammatory infiltrate in the superficial dermis	IgA ++ C3 ++
**16**	42	F	erythemapapulesvesicles	elbowskneesbuttocks	> 100	4,7	DQ2 - DR3/DQ2 - DR7	MARSH 2	Mild perivascular inflammatory infiltrate in the superficial dermis with neutrophils	IgA ++ C3 +
**17**	38	M	erythemapapulesvesicleserosion	elbowskneesbuttocks	> 100	54,7	DQ2 -DR3 *	MARSH 3A	dermal-epidermal detachment mild mixed inflammatory infiltrate in the dermis neutrophilic papilitis	IgA ++ C3 +
**18**	56	M	erythemavesiclescrust	face back elbows knees buttocks	33	41,1	ND	MARSH 3A	dermal-epidermal detachment mild mixed inflammatory infiltrate in the dermis neutrophilic papilitis	IgA ++
**19**	36	M	erythemavesicles	elbowskneesbuttocks	30	27,1	DQ8 - DR4 *	MARSH 0	Mild perivascular infiltrated in the superficial dermis dermal-epidermal detachment	IgA ++ C3 +

Anti-TG2, Anti-tissue transglutaminase-IgA; Anti-TG3, Anti-epidermal transglutaminase-IgA; HLA, Human Leukocyte Antigen; DIF, Direct Immunofluorescence; ND, Not done; *heterozygosis.

**Table 2:** Clinical features of CD patients without DH.

PT #	AGE	SEX	SERUM BIOMARKERS		HLA	MARSH
			Anti-TG2 (<9 neg 9-16 BL >16 pos)	Anti-TG3 (<16 neg 16-22 BL >22 pos)		
**1**	21	F	101	8	DQ2 - DR3 *	MARSH 3B
**2**	42	M	81,4	10,7	DQ2 - DR3 *	MARSH 2
**3**	70	M	> 100	14,4	DQ2 - DR3/DQ2 -DR7	MARSH 3B
**4**	41	M	> 100	150	DQ2 - DR3 *	MARSH 3C
**5**	43	M	25,2	79,5	DQ2 - DR3 *	MARSH 3B
**6**	30	M	35,9	3,2	DQ2 - DR3/DQ2 -DR7	MARSH 3B
**7**	45	F	94,5	25,6	DQ2 - DR3/DQ8 - DR4	MARSH 3B
**8**	48	F	> 100	6,6	DQ2 - DR7*	MARSH 3B
**9**	28	M	42,1	16,8	DQ8 - DR4/DQ7 - DR11	MARSH 3A
**10**	21	F	> 100	5,7	ND	MARSH 3 B
**11**	29	F	28*	5,9	DQ2 - DR7/DQ2 - DR7	MARSH 3A
**12**	17	M	> 100	7,3	DQ2 -DR3 *	MARSH 3C
**13**	44	F	30,4	15,2	DQ8 - DR4 *	MARSH 1

Anti-TG2, Anti-tissuetransglutaminase-IgA; Anti-TG3, Anti-epidermaltransglutaminase-IgA; HLA, Human Leukocyte Antigen; ND, Not done; * heterozygosis.

Although there is no validation, regarding a method of quantifying DH clinical severity Pemphigus Disease Area Index (PDAI) was adopted as a reasonable substitute to evaluate the extension and activity of DH (33).

The PDAI has a score ranging from 0 to 263 points, with 250 points representing disease activity (120, 10 and 120 points for skin, scalp and mucosal activity, respectively) and 13 points representing disease damage.

Specifically, PDAI has 3 components relating to the skin, scalp, and mucous membranes. The skin has activity and damage scores. The activity score value is derived from the number of erosions, blisters, or erythema and evaluates 12 anatomic locations. The individual scores are added up to provide a final activity score, which is out of 120. The damage score is 1 if post-inflammatory hyperpigmentation or erythema from a resolving lesion is present and 0 if not. A similar approach is adopted for the 12 parts of the mucous membrane score. The scalp activity score depends on the number of quadrants affected, with a maximum score of 10. The total maximum score is 263, consisting of 250 points from the activity and 13 from damage scores (33). Moreover, pruritus- Visual analogue scale (p-VAS) was used to quantify pruritus severity (34). Specifically, a 10 cm scale that at the ends has two “end points” that are defined with “no pruritus” and the “worst pruritus I can imagine”. Mild pruritus was defined as a VAS value < or =3, moderate as a VAS value between 3 and 7, and severe if the VAS score was >7. PDAI, p-VAS and serology were collected for each DH patient at first visit (T0), after 1 year of GFD (T1) and for some of them after 2 years of GFD (T2) (**Table 3**).

**Table 3:** Clinical scores of disease activity in DH patients’ follow up.

		T 0				T1				T2		
PT #	CLINICAL FINDINGS		SERUM BIOMARKERS		CLINICAL FINDINGS		SERUM BIOMARKERS		CLINICAL FINDINGS		SERUM BIOMARKERS	
	PDAI	VAS-pruritus	Anti-TG2 (<9 neg 9-16 BL >16 pos)	Anti-TG3 (<16 neg 16-22 BL >22 pos)	PDAI	VAS-pruritus	Anti-TG2	Anti-TG3	PDAI	VAS-pruritus	Anti-TG2	Anti-TG3
**1**	2	0	49	21	0	0	11	9	//	//	//	//
**2**	4	4	> 100	343	//	//	//	//	//	//	//	//
**3**	13	3	9,4	73,1	0	0	4,1	12,7	0	0	2,9	3,5
**4**	18	4	> 100	125	3	1	33,2	14,9	0	0	1,8	4,5
**5**	24	9	> 100	24,04	//	//	//	//	//	//	//	//
**6**	10	3	2,3	1,97	0	0	0,3	0,9	//	//	//	//
**7**	8	9	98	>150	0	0	9,2	75,8	0	0	9,6	11,8
**8**	10	5	15	97,3	0	0	9	11,5	//	//	//	//
**9**	8	3	> 100	281	0	0	19	5,1	0	0	3,1	6,2
**10**	14	4	>100	158	3	1	2,9	5,7	0	0	2,9	0,9
**11**	4	8	29,9	13,3	0	0	2	2,1	0	0	1,5	3,8
**12**	8	6	6,4	17,2	//	//	//	//	//	//	//	//
**13**	7	5	> 100	26,9	0	0	13,1	11,7	//	//	//	//
**14**	5	4	20	19,2								
**15**	5	9	> 100	149,7								
**16**	9	8	> 100	4,7								
**17**	21	5	> 100	54,7								
**18**	23	9	33	41,1								
**19**	15	10	30	27,1								

T0, time of the diagnosis; T1, after 1 year of gluten-free diet (GFD); T2, after 2 years of GFD; PDAI, Pemphigus Disease Area index; VAS-pruritus, pruritus- Visual Analogue Scale; Anti-TG2, Anti-tissue transglutaminase-IgA; Anti-TG3, Anti-epidermal transglutaminase-IgA.

### Direct Immuno-Fluorescence (DIF)

The presence of granular IgA deposit was analyzed in serial sections of lesional skin biopsy specimens of DH patients. A 4-mm punch biopsy was collected from all the DH patients and immediately frozen at -80°C.

The frozen specimens were cut into 5-μm thick sections and the slides were stored at –20°C until they were stained. For the staining, sections were incubated, after being brought to room temperature, with fluorescein isothiocyanate (FITC)-labelled monospecific immunoglobulins (IgG, IgA, IgM, C3; DAKO, Copenhagen, Denmark) for 30 min. Subsequently, the sections were washed in phosphate-buffered saline (PBS) for two times and mounted in buffered glycerin. Sections were visualized with Nikon C2 confocal microscope (200x) (**Supplementary Figure 1**).

### Duodenal Biopsy Examination

Three mucosal biopsies were obtained from the distal part of the duodenum of patients who were positive for anti-endomysium (EMA) and anti-TG2 antibodies. Mucosal specimens were submitted for routine histological examination according to the Marsh/Oberhuber classification (35).

### HLA Class II Typing

PB samples were collected, placed in EDTA and stored at – 20°C for DNA extraction. Genomic DNA was extracted using a commercially available kit (QIAamp DNA Mini Kit, Quiagen S.p.A., Milan, Italy). DNA was spectrophotometrically quantified, verified for purity and aliquoted at 30-40 ng/μl for molecular analysis. All patients were typed for Human Leucocyte Antigen (HLA)- DQA1, - DQB1 and - DRB1 by Real Time PCR using commercial kit (Xeligen XL, Eurospital, Trieste, Italy). In particular, haplotypes carried by celiac patients were tested searching specific alleles for HLA DQ2 – haplotype: DQA1*05, DQA1* 02:01, DQB1* 02, DRB1*03, DRB1*07, and for HLA DQ8 – haplotype: DQA1*03, DQB1* 03:02, DRB1*04. Where present, the homozygousstatus for HLA DQ2- or DQ8 – haplotype was also defined.

### Cell Culture and Flow Cytometry Reagents

The medium used was RPMI 1640 (Seromed) supplemented with 2 mM L-glutamine, 1% nonessential amino acids, 1% pyruvate, 2x10-5 M 2-mercaptoethanol (2-ME; all from Invitrogen), and 10% fetal cow serum. FITC-, PE-, allophycocyanin (APC), peridin chlorophyll protein (PerCP), Pacific Blue – conjugated anti-CD3, CD4, CD8, CD161, -IFN-gamma, -IL-4, -TNF-α were from BD Bioscience. Anti – IL-17 mAb was obtained from eBioscience, and anti- CRTH2, -CCR6 were from Miltenyi, CXCR3 was from R&D Systems. PMA, ionomycin, brefeldin A and Collagenase A were purchased from Sigma-Aldrich. rIL-2 (Proleukin^®^) from Novartis, the polyclonal activator phytohemagglutinin (PHA) from Biochrom AG, and the recall antigen streptokinase (SK) from CSL Behring; rh TG2 and TG3 were purchased from Zedira.

### Peripheral Blood and Tissue Biopsies Mononuclear Cells Recovery and Culturing

Mononuclear cells suspensions from peripheral blood (PBMNCs) of DH and CD patients were obtained by centrifugation on Ficoll-Hypaque gradient. In order to obtain tissue infiltrating mononuclear cells (MNCs), tissue biopsies from both gut and skin were enzymatically digested: they were incubated with 1 mg/ml of Collagenase A, under a gentle shaking, for 1 h at 37°C and 5% CO_2_. A further incubation of 30’ with 0.5 mg/ml of Collagenase A was then applied. Detached cells were recovered after scraping the digested tissue on a 40 μM cell strainer filter. After MNCs count in Neubauer chamber, cells were freshly evaluated by flow cytometry and/or polyclonally expanded. As most of tissue-derived samples contained very low numbers of MNCs and in order to obtain sufficient number of cells to perform phenotypic and functional evaluations we decided to expand recovered cells. To this end, tissue-derived MNCs and PBMNCs were plated on irradiated (9000 rad) allogeneic PBMCs as feeder cells (in a ratio 1:1 for PBMNCs, and on 10^5^ feeder cells for tissue-derived MNCs), in the presence of PHA, and rhIL-2 (50 U/mL). Cell culture was then expanded and rhIL-2 added every 3-4 days, for a maximum of 2 weeks.

### Phenotypical and Functional Flow Cytometric Profiling of T Cells

Membrane expression of receptors and cytokines production ability were evaluated at single cell level by flow cytometry as previously described (36). Briefly, T cells were stimulated with phorbol 12-myristate 13-acetate (PMA) plus ionomycin, Brefeldin A (BFA) was added after 2h stimulation and incubated for further 4 hours, then cells were fixed in formaldehyde and analysed for intracellular cytokines production on a BDLSR II flow cytometer (BDBiosciences). Flow cytometric data were analysed by FacsDIVA software (BDBiosciences).

### Antigen Specific T Cell Lines Generation and Testing

Short term antigen-specific T cell lines (TCLs) were generated by culturing 1x10^6^ PBMC in the presence of TG2 or TG3 (5 ug/ml) for six days; successively, activated T cells were expanded for further7- 8 days by the addition of rIL-2 (25 U/ml) every three/four days. The specificity of short-term TCLs was assessed by a thymidine incorporation assay after culturing them for 5 days with the antigen, in the presence of autologous irradiated MNCs (in a ratio TCLs vs. MNC of 1:2). TG2-induced TCLs were tested toward their own inducing antigen and also toward TG3 and a negative control, and vice-versa for TG3-induced TCLs. Stimulation index (SI) was calculated as the ratio between the c.p.m. measured in stimulated vs. unstimulated cultures. Short term antigen-induced TCLs were considered specific for a SI > 3.

### Antigen Specific T Cell Clones Generation and Testing

T cell blasts from cross-reactive antigen-specific T cell lines were cultured under limiting dilution (0,3 cell/well) in the presence of 10^5^ irradiated (9,000 rad) allogeneic PBMCs as feeder cells, 1% phytohemagglutinin (PHA), (vol/vol), and 50 U/ml rIL-2, in order to obtain T cell clones. T cells cultures were diluted 1:2 and added with feeder cells plus rhIL-2 every week for 4 weeks. The specificity of T cell clones (TCCs) was assessed by a thymidine incorporation assay after culturing them for 4 days with the antigen, in the presence of autologous irradiated MNCs (in a ratio TCCs vs. MNC of 1:2). TCCs ability to proliferate in response to their cognate antigen was tested in the presence of TG2, TG3 and streptokinase (SK) as negative control: PHA plus IL-2 was used as positive control. Stimulation index (SI) was calculated as the ratio between the c.p.m. measured in stimulated vs. unstimulated cultures. TCCs were considered specific for a SI > 10.

### TCR V-Beta Repertoire Evaluation

Flow cytometric analysis of the TCR Vβ repertoire of human T lymphocytes was performed by using the IOTest^®^ Beta Mark (Beckman Coulter) according to the manufacturer’s instructions.

### Celiac Disease-Related Antibodies

Serum samples were collected from all DH and CD patients included in our study and stored at – 20°C until they were tested. IgA anti-TG2 were measured by commercially available ELISA kit (Eu-tTG IgA, Eurospital, Trieste, Italy) using recombinant human tissue transglutaminase as antigen. According to the manufacturer’s instructions, serum was diluted 1:100, and absorbance was measured at 450 nm. The cut-off for positive values was 16 U/ml Anti-TG2-IgA antibodies. Samples were further tested for anti-Endomisium-IgA (EMA) using an indirect immunofluorescence assay with a commercially available test kit (Eurospital, Trieste, Italy).

### Statistics

The Student t test (paired and not paired) was used for flow cytometric frequencies statistical analysis. P values less than or equal to 0.05 were considered significant. Pearson’s correlation coefficients were used to calculate the correlations.

## Results

### TNFα Producing Cells Are Enriched in the Skin of Dermatitis Herpetiformis Patients

In order to compare T cell response between DH and CD patients, the following intracellular cytokines expression, upon polyclonal stimulation, were evaluated by flow-cytometry: IFNγ for Th1 and Tc1, IL-17A for Th17 and Tc17, IL-4 for Th2 and Tc2 cells and TNFα as a representative pro-inflammatory CK. The membrane expression of chemokine receptors CXCR3 and CCR6, and the surface molecules CD161 and CRTH2 was also evaluated as representative of the different T helper subsets, but relevant differences were not observed (see **Supplementary Figure 2**). Skin, gut and circulating mononuclear cells (MNCs) were *in vitro-*expanded in non-polarizing conditions for two weeks in the presence of irradiated feeder cells and phytohemagglutinin plus recombinant IL-2, due to the low numbers of cells derived from fresh samples of gut and skin in particular. With regard to skin specimens of DH patients we obtained 50x10^3^ ± 9x10^3^ MNCs, and only 14x10^3^ ± 3.9 x10^3^ MNCs from CD patients (p =0.012 data not shown) making it impossible to evaluate cytokines production on fresh cells from the CD group.

**Figure 1:**
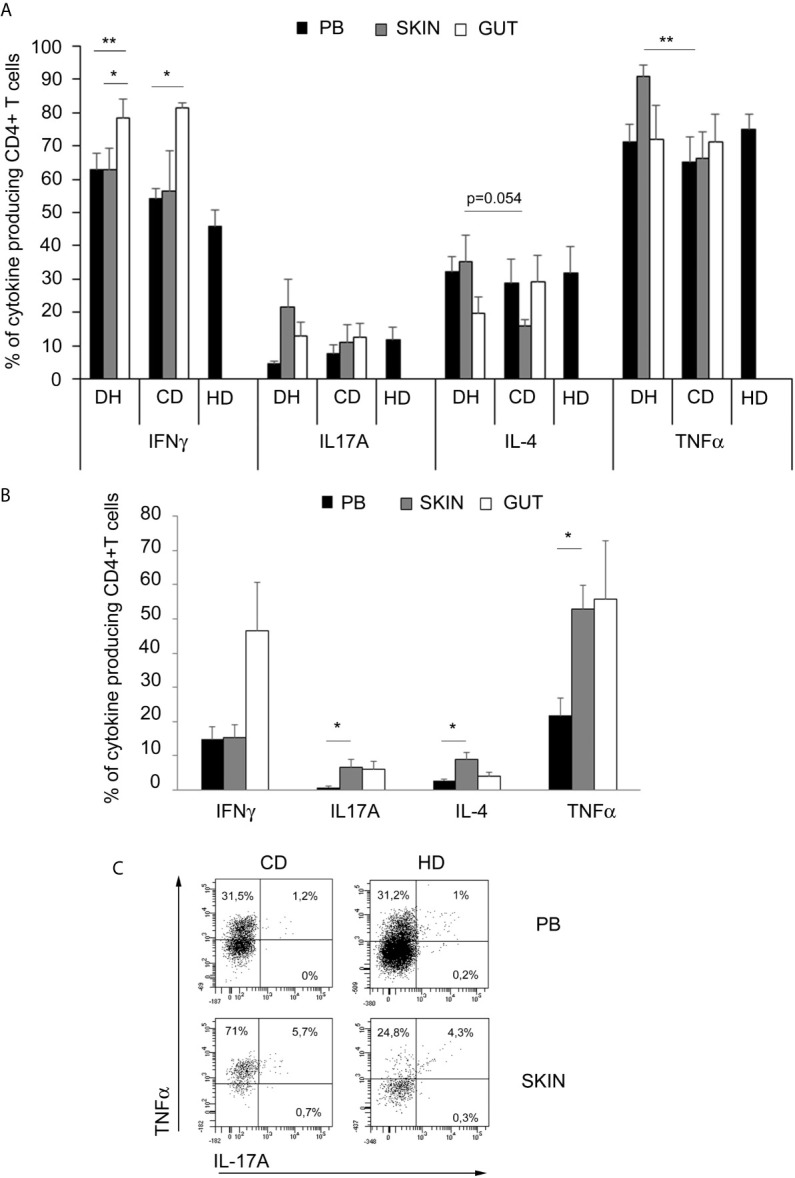
TNFα-producing cells are enriched in skin samples from DH patients. **(A)** Intracellular cytokines production in *in vitro* expanded CD4+ T cells from PB (black columns), skin (grey columns) and gut (white columns): frequencies of cytokines producing of CD4+CD3+-gated cells were assessed in 10 DH patients, 6 CD patients and 4 healthy donors. Columns represent means ( ± SE). Bars * indicate p ≤ 0.05; bars ** indicate p ≤ 0.01. **(B)** Frequencies of intracellular cytokine producing CD4+ T cells in *fresh* samples from PB (black columns), skin (grey columns) and gut (white columns) of 5 DH patients. Columns represent means ( ± SE). Bars * indicate p ≤ 0.05. **(C)** Representative flow cytometric analysis obtained in one out of five subjects with DH and in only one healthy specimen. Percentages of CD4+ T cells are shown. PB,peripheral blood.

As shown in **Figure 1A**, we found that either in DH patients and in CD patients, gut-derived T cell lines were enriched in IFNγ producing CD4+ T cells compared to peripheral blood (PB), while TNFα producing cells were significantly increased in skin samples only in patients affected by DH. Accordingly, the frequency of skin-derived CD4+ T cells producing TNFα was significantly higher in DH patients when compared to CD ones. We also found a nearly significant (p=0.054) increase in the frequency of skin derived CD4+ T cells producing IL-4 in DH than in CD patients. Concerning the frequency of IL-17 producing CD4+ T cells, we observed a trend to be higher in the skin of DH patients when compared to PB. No appreciable differences raised from the comparison with the frequency of cytokine producing TCLs deriving from PB of healthy donors. Results on CD8+ T cells only showed that Tc1 cells were slightly but significantly increased in the gut of DH patients when compared to CD ones (data not shown). Finally, the chemokine receptor expression on both CD4+ and CD8+ T cells resulted comparable among the different tissues (**Supplementary Figure 2**).

In order to exclude that the observed data were a result of an *in vitro* artefact, due to the generation of short- term polyclonal T cell lines, we dedicated all the cells deriving from skin, gut and PB samples from 5 subsequent DH patients to the cytokines production evaluation on freshly isolated lymphocytes. As shown in **Figure 1B**, the data confirmed those obtained on T cell lines: IFNγ producing CD4+ T cells resulted enriched in the gut while TNFα producing cells were mainly enriched in the skin. Interestingly, the trends of IL-4 and IL-17A producing CD4+ T cells to be increased in skin samples, compared to PB, were confirmed by the ex-vivo analysis. Freshly isolated CD4+ lymphocytes from the gut mucosa of DH patients seemed to be enriched in TNFα producing cells compared to PB ones but without statistical significance. Of note, we had the opportunity to evaluate fresh MNCs obtained from a large specimen of healthy skin, about 14 cm^2^, deriving from a subjects underwent to mastectomy before any chemo- or radio-treatment. Interestingly, the frequency of TNFα producing CD4+ T cells resulted similar to the one of PB and much lower than that observed in skin of DH patients.

### The Frequency of Circulating TNFα and IL-17A Producing T Cells Positively Correlates With Disease Activity Scores and Reduces After Gluten Free Diet in Dermatitis Herpetiformis Patients

**Figure 2:**
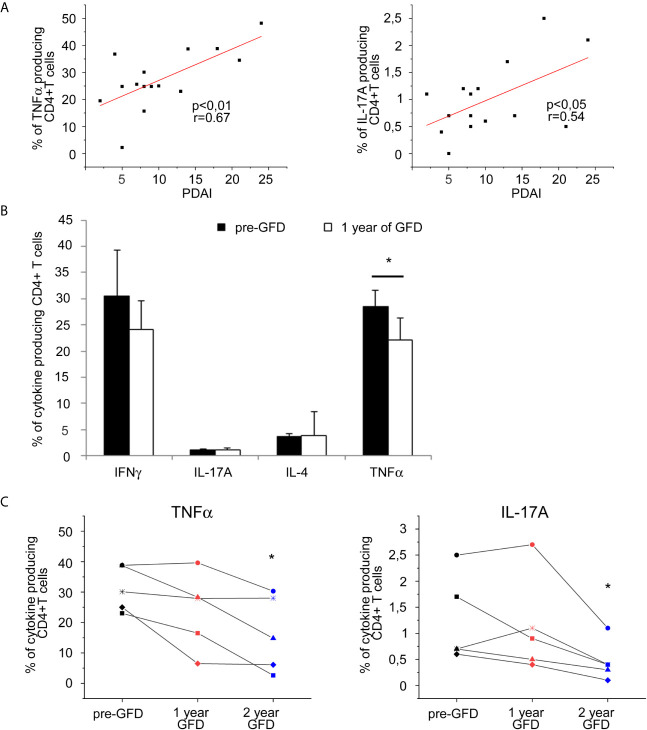
PB Cytokine-producing T cells frequencies correlation with disease activity score and evaluation after gluten free diet in DH patients. **(A)** Correlation between disease activity score PDAI and frequencies of circulating CD4+ T cells producing TNFα (left panel) and L-17A (right panel) was calculated in 15 DH patients. **(B)** Frequencies of intracellular cytokines producing CD4+ T cells in *fresh* samples from PB were evaluated in 8 DH patients before and after 1 year of gluten free diet (GFD). Columns represent means ( ± SE) of the indicated cytokine. *p ≤ 0.05. **(C)** Frequencies of circulating CD4+ T cells producing TNFα (left panel) and IL-17A (right panel) in *fresh* samples from PB were evaluated before (To) and after 1 and 2 year of gluten free diet (GFD) in 5 DH patients. ( ± SE). *p ≤ 0.05 compared to the To evaluation.

Searching for further evidences about cytokines involvement in DH pathogenesis, we correlated the frequencies of cytokine producing CD4+ T cells from DH patients’ specimens with the clinical score of disease activity Pemphigus Disease Area Index (PDAI). Anyway, frequencies of TNFα producing CD4+ T cells in skin derived cell lines resulted systematically extremely high making not possible to observe any clear correlation trend (data not shown); thus we moved to the study of freshly isolated circulating lymphocytes. Interestingly, as shown in **Figure 2A**, we found a positive correlation of PDAI with the frequencies of circulating CD4+ T cells producing TNFα and also with those producing IL-17A, in harmony with the previous data. Accordingly, when evaluating PB samples before and after one year of GFD, we discovered that circulating TNFα producing CD4+ T cells significantly decreased after gluten free diet associating to the clinical amelioration of skin involvement (**Figure 2B**). Moreover, we longitudinally monitored 5 DH patients along 3 years: results are summarized in **Figure 2C** and show that after 2 years of GFD TNFα producing cells keep decreasing and even IL17-producing CD4+ T cells became significantly reduced at the second year of follow-up.

### Dermatitis Herpetiformis and Celiac Disease Patients’ T Cells Showed a Crossed Proliferative Response to Transglutaminases

**Figure 3:**
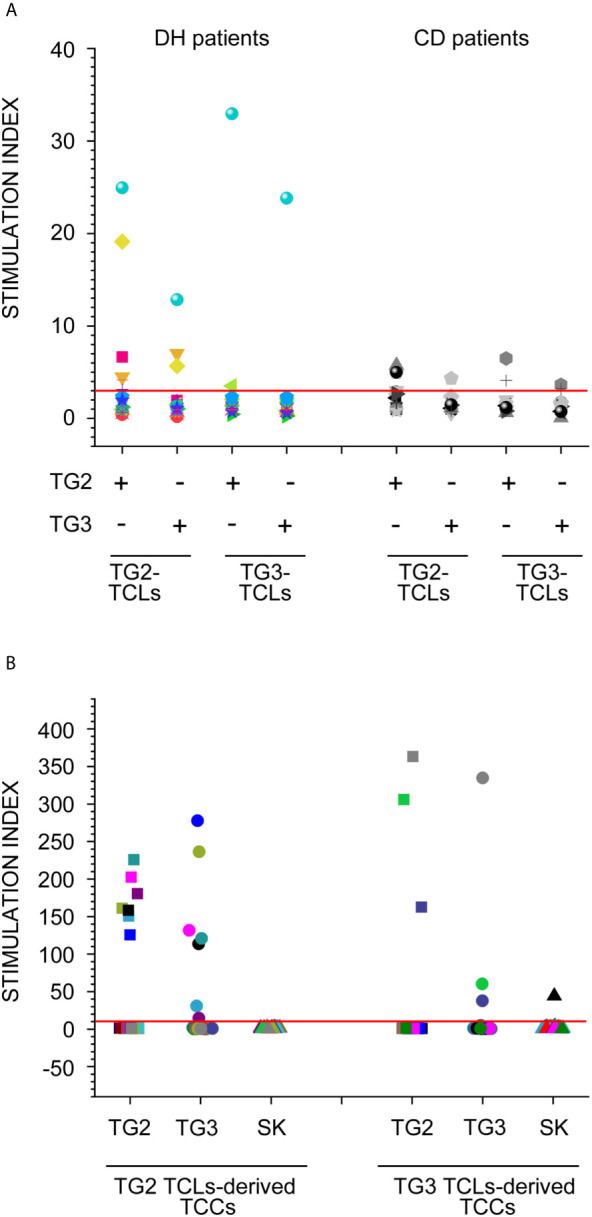
Transglutaminases-specific T cells proliferation assays. **(A)** Proliferation assay was performed on PB derived TCLs, induced in the presence of TG2 and TG3, from 15 DH patients (left panel) and 12 CD patients (right panel). Each TCLs was stimulated with/without both TG2 and TG3 antigens. Dots with the same shape and colour represent stimulation index (SI) of TCLs derived from the same patients. **(B)** Proliferation assay was performed on T cell clones (TCCs), derived from antigen specific TCLs from 1 DH patients. Each TCC was stimulated with/without both TG2 and TG3 antigens as well as streptokinase (SK) as negative unrelated control. Dots with the same colour represent SI of the same TCC.

In addition to the phenotypic assessment of T cell response, we also investigated the antigen specificity of circulating T cells obtained from DH and CD patients toward tissue and epidermal transglutaminases (TG2 and TG3 respectively). To this end, we obtained transglutaminase-specific TCLs by culturing PBMNCs from CD and DH patients as described in material and methods. Thereafter, we verified the specificity of TCLs by further 4 days of culturing in the presence of autologous irradiated PBMNCs, as antigen presenting cells, and the inducing antigen (i.e. TG2) as well as the other isoform of transglutaminase (i.e. TG3) to test possible cross-reactions. We also used streptokinase (SK) as uncorrelated control antigen. Results are represented in **Figure 3A** and show that TG2 specific TCLs have been obtained on both DH and CD patients, with a tendency of a higher reactivity in DH patients, while TG3 resulted less immunogenic. We also found that some TCLs proliferated in response to both TG2 and TG3 in the two groups of patients. In order to verify if this result was actually due to a cross-reactive T cell response against TG2 and TG3 or to the presence in the same cell culture of different T cells with distinct TCR specificities, we derived T cell clones starting from cross-proliferating TCLs from 1 DH and 1 CD patient. All the T cell clones responding to TG2 also responded to TG3 and vice versa (**Figure 3B**), confirming a real cross-reactivity between the two autoantigens. Unfortunately, from CD patient, we obtained a very low number of T cell clones, probably due to the low proliferative rate of the originating TCLs. However, despite the low cell recovery from CD patients, cross-reactivity was evident between TG2- and TG3- reactive clones. (Data not shown). Finally, we confirmed the real single clone origin of the above-mentioned cross-reacting T cell clones by flow cytometric analysis of TCR Vβ repertoire (**Supplementary Figure 3**).

## Discussion

Dermatitis herpetiformis (DH) is considered the cutaneous manifestation of celiac disease (CD). Both diseases are triggered by gluten, the necessary presence of HLADQ2/DQ8 genetic profile, and the generation of circulating autoantibodies to tissue transglutaminase (TG2). Due to the lymphocytic infiltration observed in the active CD intestinal lesion (6), and the strong association of the disease with HLA molecules (7), the concept of T-cell involvement in the pathogenesis of CD has been long established prior to the isolation of gliadin-specific T cells from the CD intestinal mucosa (8). In this context, TG2 is one of the autoantigens involved in CD, and it also plays a role through the deamidation of certain gluten peptides, increasing their affinity to HLA-DQ2 and/or HLA-DQ8. This process generates a more vigorous CD4+ T-helper cell activation, which can result in intestinal mucosa inflammation, symptoms of malabsorption, and secondary extra intestinal manifestations such as DH. In this heterogeneous scenario, why only a subgroup of patients suffering with CD will develop DH, and why the cutaneous involvement lasts longer than the intestinal one, after the introduction of a gluten-free diet, is still unclear. Moreover, while the intestinal immune response to gluten has been widely studied either in terms of helper T cell subsets involvement or of antigen specificity (10, 37), the same did not occur for DH. Recently, Th17 cells acquired an increasing role in immune-mediated diseases; this subpopulation of T helper lymphocytes is characterized by a high degree of plasticity, that is the ability to shift its phenotype, in presence of specific micro environmental stimuli, toward the Th1 phenotype (Th17/Th1) (38, 39). The involvement of different T helper sub-populations in the development of DH has been hypothesized only in few preliminary manuscripts (25). In the present work we aimed to assess the T helper equipment in patients suffering with DH both by evaluating the production of the cytokines related to the main T helper cell subpopulations (Th1, Th2, Th17), and by the assessment of T cell antigen specificity. The phenotypical features were assessed on T helper cells infiltrating the skin, the gut and present in peripheral blood. The comparison between the results obtained from the three tissues in DH patients *vs.* coeliac patients without skin involvement, showed a significant increase in CD4+ T cells producing TNFα at skin level that selectively occurs in DH patients. Moreover, in freshly evaluated samples also IL-17A- and IL-4-producing lymphocytes resulted significantly increased in the skin, compared to PB in DH patients. These results are in agreement with published data describing a key role of neutrophils and eosinophils in the induction of skin damage in DH (18). Thus, the two pro-inflammatory cytokines TNFα and IL-17A seem to be a signature of the skin involvement during DH manifestation. To strengthen the hypothesis of a relevant role of these two cytokines in DH pathogenesis, we attempted to correlate them to the clinical activity of skin involvement. Since there is no validation regarding a method of quantifying DH clinical severity, PDAI was adopted as a reasonable substitute for this purpose. Interestingly, a positive and significant correlation between TNFα and IL-17A and the clinical score PDAI was observed in DH patients. The possible association between pro-inflammatory cytokines producing cells at peripheral blood level, and the active phase of skin involvement in DH patients, was also investigated by evaluating the cytokine production ability of circulating T helper lymphocytes, 1 and 2 years after gluten free diet. A significant and progressive decrease of the frequency of TNFα-producing cells was observed after the first year of gòuten free diet, and a further decrease was appreciated after the second year. A similar trend was appreciated also for IL-17 producing cells frequencies, but in this case a significant reduction was evident only after the 2^nd^ year of gluten free diet.

While the first part of the work was dedicated to the study of the T helper cell subsets, and their possible involvement in DH pathogenesis, in the second part of the work we aimed to evaluate the antigen specificity of T cells. To this end, we considered T cell proliferative response toward TG2 and TG3, the two main auto-antigens involved in CD and in DH, respectively. In particular, on the basis of the relatively high degree of homology between the two auto-antigens (about 65%) (40), we hypothesized a possible cross-reaction between these antigens. To address this point we obtained TG2 and TG3 antigen-specific T cell lines from the peripheral blood of both DH and CD patients. The first data obtained is that the frequency of antigen specific TCLs and the intensity of T cell proliferation upon TG2 or TG3 stimulation tend to be higher in DH respect to the CD counterpart. More interestingly, TG2-specific TCLs, obtained from the DH group, resulted cross-reactive with TG3 and the only one TG3-specific TCL, was highly cross-reactive with TG2. Even among CD patients TCLs were cross-reactive (1 among TG2-specific and 1 in TG3-sepecific TCLs) but their stimulation index were lower compared to those of DH ones and borderline to the cut off value. The differences between DH patients and CD ones, concerning both the number of specific T cell lines obtained and the intensity of T cell proliferation rate to antigen triggering, does not reach the statistical significance, due to the low number of these kind of experiments. This was depending on the rarity of DH patients and on the technical difficulty to obtain TG2- and TG3- specific T cell lines. For this reason, this part of our result must be considered still preliminary, and necessitate of further confirmation on a larger cohort of DH and CD patients. However, the observed higher frequency of cross reactive TCR in DH patients compared to CD ones, if confirmed by further works, could suggest the reason why only some CD patients develop DH. Since TCLs are by definition oligoclonal and not monoclonal cell cultures, we could not assume that the cross-reaction we observed between TG2 and TG3 was based on a real cross-reactivity of two different antigens recognized by one single TCR. For this reason, we confirmed such cross-reactivity even on T cell clones model. These data provide new insight in the biological mechanisms that drive CD patient to develop also DH: they suggest that a certain number of TG2-specific T cells, very likely originating from the gut associated lymphoid tissue (GALT), displaying a clonal expansion in course of CD, are also specific for TG3. These GALT-deriving T cells are thus potentially responsible for skin involvement. Of note, TG2/TG3 antigen specific lymphocytes can be obtained also by CD affected patients in the absence of clinical DH manifestation. This evidence points out that virtually all CD subjects display the potential risk to develop DH, even if in the majority of CD patients the clinical manifestation of DH does not occur. Probably the presence of some regulatory mechanisms downstream the TCR/antigen recognition, prevents the development of the skin involvement in celiac patients. It is likely that a crucial role could be played by a defect in immune regulation or, on the other hand, could be pivotal the activation of some skin homing pathways for T cells and/or the skewing of T helper cells toward a pathogenic phenotype. Several recent papers investigated and hypothesized different immune-pathogenesis mechanisms for DH (32). Antiga E. et al. showed a lower proportion of FOXP3(+) Tregs and IL-10(+) cells in skin of DH patients than in healthy subjects: this could lead to a defect on suppressive function explaining the previous described exceeding T helper cell response (25). Few year later Hall RP III et al. found an increased expression of E-sel mRNA in the normal-appearing skin of patients with DH associated with increased serum levels of sE-sel, IgA anti-tissue transglutaminase (TG2), serum IL-8, and serum TNFα. These results suggest that the presence of an active mucosal inflammatory response in the gut of patients with DH is associated with an activation of cutaneous endothelial cells as well as circulating inflammatory cells: these processes may play a key role in the recruitment of T cells in the skin of DH patients (41). Finally, a recent paper reported that local skin production of cytokines and chemokines, including TNFα and IL-17A, in the presence of TG3/IgA deposits allows migration of adherent neutrophils to the papillary dermis, inducing a sub epidermal split by cleaving adhesion molecules of the basement membrane zone, such as collagen VII (42). In conclusion, the present study provides new insight concerning the immunological bases of DH, both at level of auto-antigen recognition and T helper cells phenotype. In particular, our results demonstrate the existence of a clear T cell cross-reactivity between TG2 and TG3, as a basis for the T cell recognition of TG3, the crucial autoantigen in DH. Moreover they suggest the involvement of a Th17 skewed response at skin level during DH manifestation, with a marked pro-inflammatory feature, orientated by TNFα production. These data offer a new scenario in DH pathogenesis and open new possible therapeutic strategies in the field of immunotherapy, oriented to block TNFα or IL-17A. This is particularly important in those patients with severe skin exacerbation during the period of latency before the clinical response to the diet, as well as in refractory patients.

## Data Availability Statement

The original contributions presented in the study are included in the article/**Supplementary Material**. Further inquiries can be directed to the corresponding author.

## Ethics Statement

The studies involving human participants were reviewed and approved by Azienda USL Toscana Centro, P.O. Piero Palagi, 663/2013/OSSCESM. Written informed consent to participate in this study was provided by the participants’ legal guardian/next of kin.

## Author Contributions

FA, FL, MarC, and AC designed research studies. ManC, VS, MR, AM, LM, BR, GL, BB, and DR conducted experiments. ManC, LQ, and GL acquired data. ManC, FL, MarC, and LQ analyzed data. GL, MarC, and LQ collected Peripheral blood samples and acquired informed consent. ManC, MarC, FL, LQ, DR, and AC wrote the manuscript. All authors contributed to the article and approved the submitted version.

## Funding

The study was funded by a Grant from the Foundation for Celiac Disease of The Italian Society for Celiac Disease, within the funding program FC_2014 (project code: 017_FC_2014) and by a fellowship from the Foundation for Celiac Disease of The Italian Society for Celiac Disease, within the funding program FC_2016 (project code: 003_FC_2016).

## Conflict of Interest

The authors declare that the research was conducted in the absence of any commercial or financial relationships that could be construed as a potential conflict of interest.

## Supplementary Material

Supplementary Figure 1 Direct immunofluorescence image of dermal biopsy of a DH patient. Granular IgA deposits (red arrows) at the dermal papillae and along dermal-epidermal junction detected on perilesional skin specimen from patient suffering with DH (Direct Immunofluorescence magnification 200x).

Supplementary Figure 2 Membrane expression of T helper subsets’ specific surface molecules and chemokine receptors Membrane expression of CXCR3 (representative of Th1 cells), CCR6 and CD161 (representative of Th17 cells) and CRTH2 (representative of Th2 cells) in *in vitro* expanded CD4+ T cells from PB (black columns), skin (grey columns) and gut (white columns). Frequencies of CD4+ T cells were assessed in 7 DH patients, 7 CD patients and 4 healthy donors. Columns represent means ( ± SE).

Supplementary Figure 3 Single clone nature of cross-reacting T cell clones (TCCs), Representative dot plots of TCR Vβ Repertoire analysis on 2 TCCs cross-proliferating to TG2 and TG3: in the upper panel of the figure is shown the analysis on a TCC from a DH patient and in the lower panel one from a CD patient.
