# Supplementary figures and images for: T Cell Response Toward Tissue-and Epidermal-Transglutaminases in Coeliac Disease Patients Developing Dermatitis Herpetiformis
Source: Front Immunol. 2021 Apr 20;12:645143. doi: 10.3389/fimmu.2021.645143 (PMC8093623; doi:10.3389/fimmu.2021.645143)

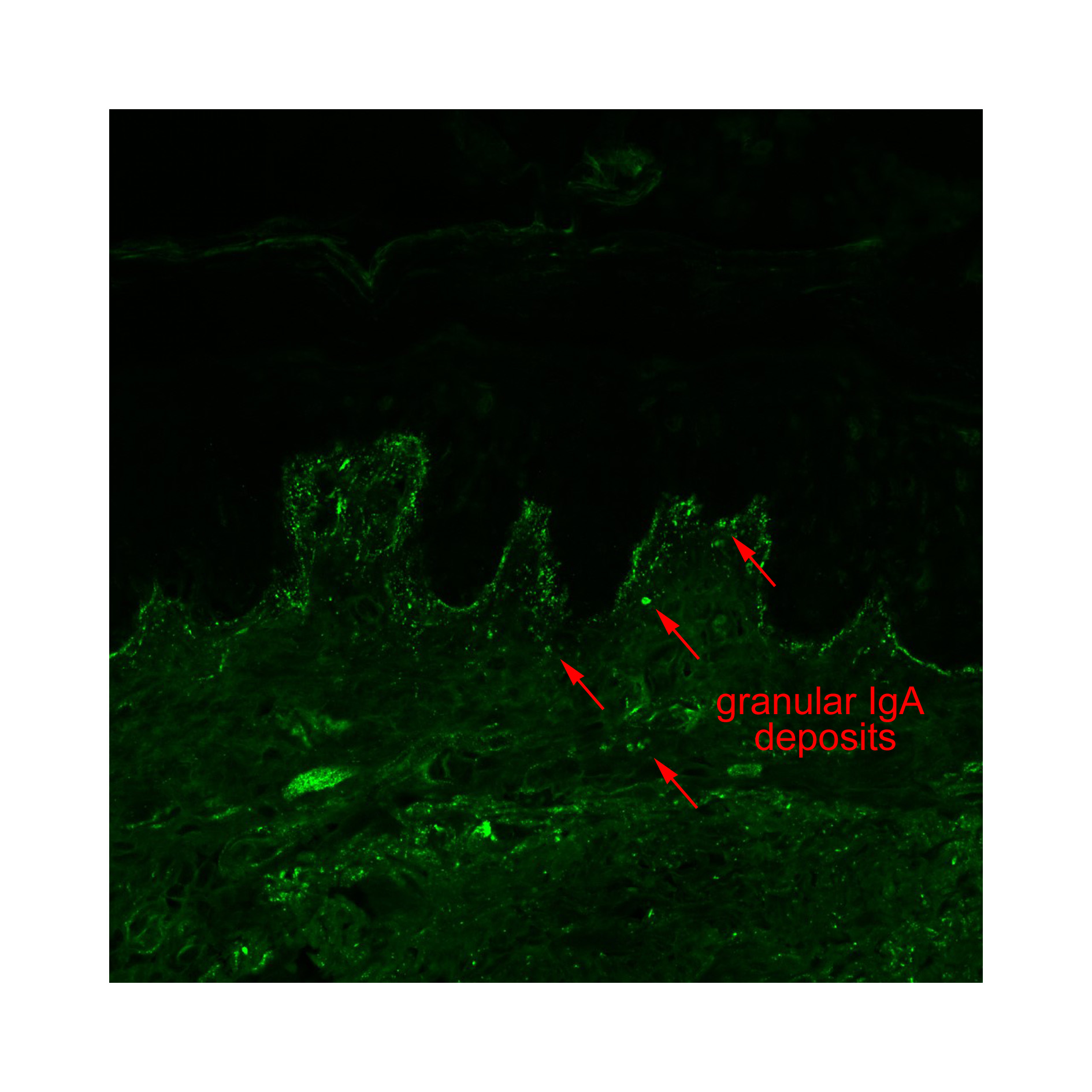

Supplement: Supplementary Figure 1 — Direct immunofluorescence image of dermal biopsy of a DH patient. Granular IgA deposits (red arrows) at the dermal papillae and along dermal-epidermal junction detected on perilesional skin specimen from patient suffering with DH (Direct Immunofluorescence magnification 200x). [file Image_1.jpeg]

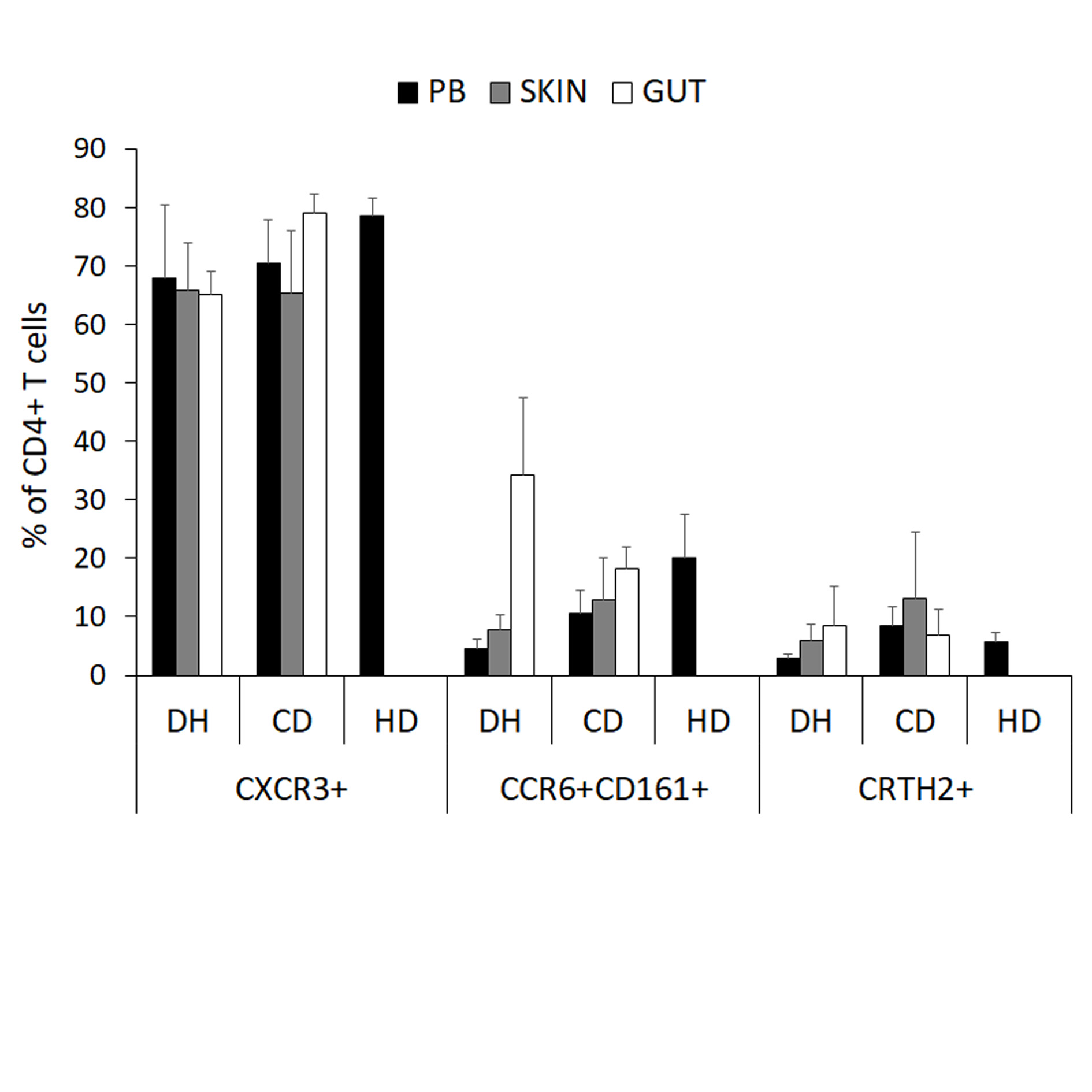

Supplement: Supplementary Figure 2 — Membrane expression of T helper subsets’ specific surface molecules and chemokine receptors Membrane expression of CXCR3 (representative of Th1 cells), CCR6 and CD161 (representative of Th17 cells) and CRTH2 (representative of Th2 cells) in in vitro expanded CD4+ T cells from PB (black columns), skin (grey columns) and gut (white columns). Frequencies of CD4+ T cells were assessed in 7 DH patients, 7 CD patients and 4 healthy donors. Columns represent means ( ± SE). [file Image_2.jpeg]

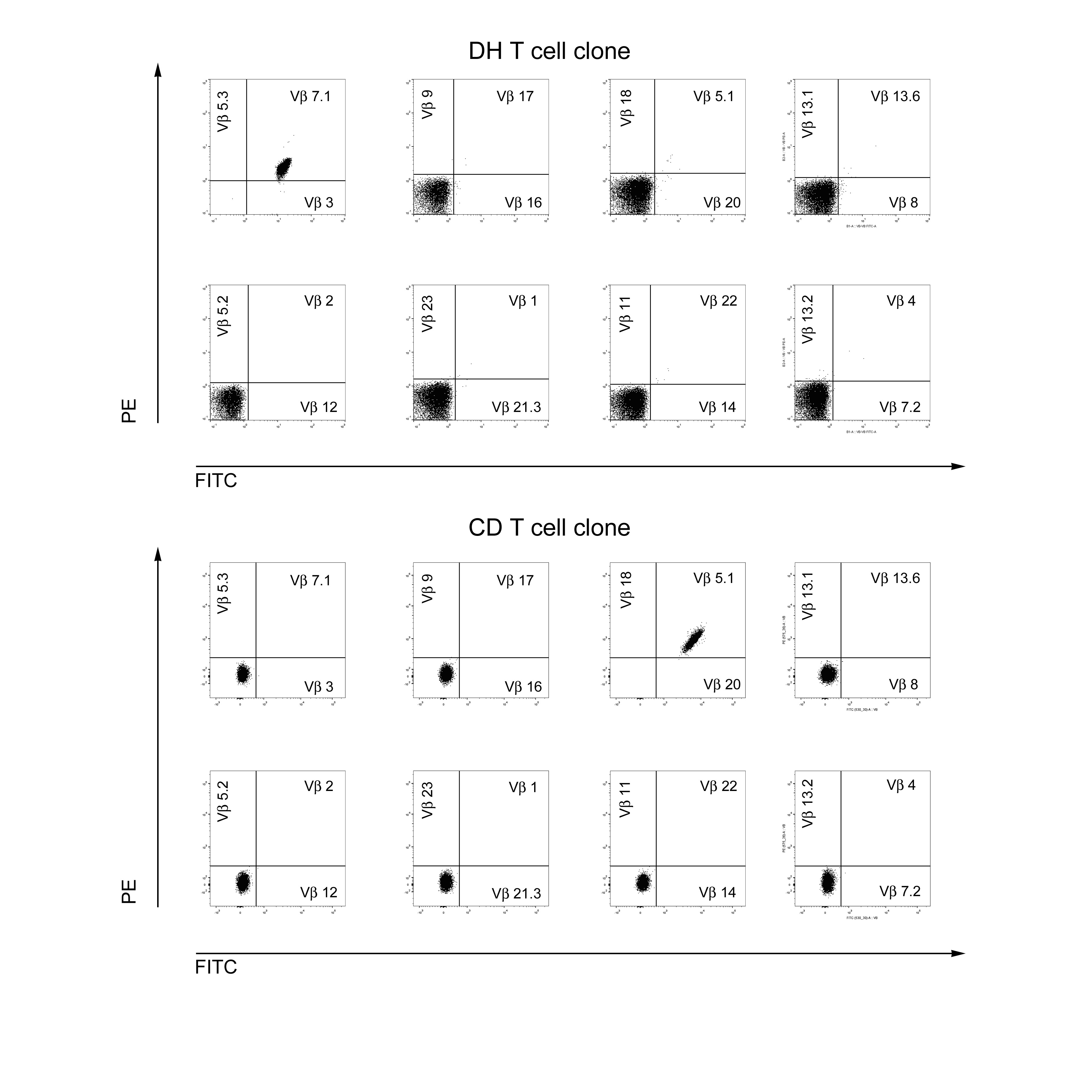

Supplement: Supplementary Figure 3 — Single clone nature of cross-reacting T cell clones (TCCs), Representative dot plots of TCR Vβ Repertoire analysis on 2 TCCs cross-proliferating to TG2 and TG3: in the upper panel of the figure is shown the analysis on a TCC from a DH patient and in the lower panel one from a CD patient. [file Image_3.jpeg]
